# Exploring the therapeutic potential of H1-antihistamines in endometriosis—A gene regulation-based perspective

**DOI:** 10.3389/fmed.2025.1538368

**Published:** 2025-07-17

**Authors:** Kameswara Bharadwaj Mantha, Mohan Kumar Gajendran

**Affiliations:** ^1^University of Minnesota, Twin Cities, Minneapolis, MN, United States; ^2^School of Science and Engineering, University of Missouri-Kansas City, Kansas City, MO, United States

**Keywords:** endometriosis, endometrium, gene expression, inflammation, H1-antihistamines

## Abstract

**Introduction:**

Recent studies emphasize the role of immune dysregulation and inflammation in endometriosis (ES). While hormonal therapy remains the primary treatment, emerging research is exploring synergistic approaches that target inflammation. In this study, we investigate the potential of H1-antihistamines (H1-As) in ES management from a gene-regulation viewpoint.

**Methods:**

We perform differential gene expression analysis on two gene-sequencing datasets from ES patients, with a primar focus on inflammatory signaling [nuclear factor-kappa B (NF-κB), tumor necrosis factor (TNF), and cytokine–cytokine receptor] and histamine synthesis and metabolism (HSM) pathways, considering disease severity and hormonal therapy usage.

**Results & Discussion:**

Consistent with the literature, our findings highlight the dysregulation of several genes involved in pro-inflammatory pathways, including interleukins (ILs), cyclooxygenase-2 (COX-2), chemokine ligands, cellular adhesionmolecules, and neuroangiogenesis. We also note dysregulation of genes in the HSM pathway, indicative of a microenvironment that favors histamine availability and inflammatory persistence through enhanced histamine synthesis and reduced breakdown, as well as a reduced potential to clear reactive aldehyde species. We also find that hormonal therapy minimally affects the dysregulation of the majority of pro-inflammatory and histaminic pathway genes, and their amplified dysregulation is noted in early stage disease. By placing our findings in the context of existing evidence on histamine-mediated modulation of inflammatory pathways via the H1 histamine receptor (HRH1), we present a comprehensive discussion on the potential therapeutic value of H1-As in ES management due to their anti-inflammatory and mast-cellstabilizing properties.

## 1 Motivation

Endometriosis (ES) is a condition characterized by the estrogen-dependent ectopic growth and proliferation of endometrial cells outside of their intended eutopic uterine environment. This process induces chronic inflammation and leads to chronic pain and infertility in reproductive-age women. It is estimated that ~5% of premenopausal women suffer from ES (1), with a peak incidence among women in the age group 25–35 years (2), with an especially amplified incidence (~50%) among those undergoing fertility treatments (3, 4). ES is associated with a plethora of burdening symptoms (5–7) such as chronic pelvic and referred pain, irregular and excessive menstrual bleeding, psychological burden, and infertility. However, ES often remains underdiagnosed with an average delay from symptom onset to diagnosis of ~7–10 years (8), owing to its relatively non-specific symptoms and radiological findings during earlier disease stages.

In the literature spanning almost a century, various hypotheses have been proposed on the pathogenesis of ES. Some commonly cited theories include the retrograde menstruation process, where eutopic (shedding) endometrial cells are transported to the extrauterine space; the coelomic metaplasia theory, which posits that the transformation of extrauterine, non-endometrial tissue into an endometrial-like lining; and immune system dysregulation causing an impaired “clearance” of ectopic tissue. See Lamceva et al. (9) for a comprehensive review of various theories. Depending on the incidence of endometrial lesions, ES is referred to using specific subtypes (see International Working Group of AAGL et al. (106)) such as peritoneal ES (i.e., comprising superficial peritoneal lesions), ovarian ES (comprising ovarian endometriomas), deep infiltrating ES, and various other organ-specific ES (e.g., bowel, rectovaginal, bladder, etc.).

Surgical resection of ES lesions and associated peritoneal adhesions is often the initial step in both confirming the diagnosis and initiating a long-term therapeutic regimen (10). Pharmacological management of ES and associated symptoms predominantly involves the use of hormonal therapies that often target the suppression of estrogen synthesis, thereby halting the necessary proliferation signaling to the endometrial cells [see review by Mitranovici et al. (11)]. A range of options has been approved for use in ES, starting with estrogen–progesterone combinations or progesterone-only pills, injectable or implantable long-acting progestin devices, gonadotropin-releasing hormone (GnRH) agonists and antagonists, often co-managed with general non-steroidal anti-inflammatory drugs (NSAIDs) or specific cyclooxygenase-2 (COX-2) inhibitors. Novel options are also under experimental consideration (e.g., selective estrogen and progesterone receptor modulators).

Through the insights from various systemic, local, and gene-level studies, the complex role of the immune system and pro-inflammatory microenvironment in the pathogenesis of ES is increasingly becoming evident and recognized (12, 13). Several studies have identified the dysregulation and enhanced activation of pro-inflammatory signaling cascades [e.g., involving nuclear factor-kappa B (NF-κB), tumor necrosis factor alpha (TNF-alpha)] (14, 15), potentiated by recruitment and activation of macrophages and mast cells. Such a scenario facilitates the sustainability of ES lesions through complex cytokine and chemokine signaling [e.g., interleukins (ILs), IL-1β, IL-6, and IL-8; TNF-alpha] (16), enhanced cellular adhesion (17–19), and neuroangiogenesis (20). Given the crucial role of inflammation in ES, active research is underway to find novel therapeutic agents that specifically target such pathways.

H1-antihistamines (H1-As) are a class of well-studied drugs that act through inverse agonism of H1 histamine receptor (HRH1) and have been widely used in treating allergic and reactive conditions involving the pulmonary and dermatological systems (21). Several studies that charted H1-As main action pathways highlight their role in imparting systemic anti-inflammatory and immunomodulatory action (22, 23). In our recent evidence-based perspective (24), we highlighted the commonalities shared by dysregulated pro-inflammatory pathways in ES and those that are preferentially targeted and countered by H1-As. As such, we proposed H1-As as a potential therapeutic option for the management of ES.

Building on the aforementioned perspective from Mantha (24), through this quantitative study, we explore the regulation of genes participating in specific inflammatory signaling (NF-κB, TNF, and cytokine–chemokine signaling) and histamine synthesis and metabolism (HSM) pathways using the latest and largest high-throughput sequencing data of endometrial tissues from patients with and without ES. By assessing the up- and downregulation of genes participating in these pathways, supported by a broader perspective and a discussion on the role of histamine in modulating inflammation, we present supportive arguments advocating for the need to study H1-As in the context of ES further.

This study is structured as follows: Section 2 presents the datasets used in this work, and Section 3 outlines our methodological details related to differential gene expression (DGE) analysis. In Section 4, we present the DGE analysis results for each of the two datasets used in this work. In Section 5, we discuss and compare our DGE analysis results with those from other literature, and summarize our quantitative conclusions in Section 6. In Section 7, we present a more comprehensive discussion from the perspective of histamine and antihistamine action in the context of existing therapeutic options for ES. We provide concluding remarks that highlight the need for focused studies to consider H1-As as a synergistic therapeutic option for ES management, offering broader implications.

## 2 Data

In this study, we use two gene sequencing datasets from the Gene Expression Omnibus (GEO): GSE141549 (25) (i.e., EndometDB database) and GSE51981 (26). Here, we provide a brief overview of these datasets and the subcategorization of ES-patient samples based on disease severity, tissue type, menstrual cycle phase, medication status, and other relevant factors.

### 2.1 GSE141549 (EndometDB)

The EndometDB dataset comprises gene sequencing data from 168 samples−115 ES patients and 53 disease-free controls. This data is further augmented with various clinical features such as age, disease stage, tissue/lesion type, menstrual cycle phase, and use of hormonal medication [see Figure 2 in Gabriel et al. (25)].

Briefly, the samples' menstrual cycle phases are indicated as proliferative, secretory, or undetermined owing to medication usage. The acquired tissue/lesion types are categorized as follows: Control endometrium (CE) and patient endometrium (PE), control Peritoneum (CP) and patient peritoneum (PP), peritoneal lesions (PeLs), and deep infiltrating ES (DiE) subdivided as per the site of involvement: bladder, intestinal, rectovaginal, sacrouterine ligaments, and ovaries.

In this work, we perform DGE analysis on the EndometDB dataset by combining the different categories into specific subsets as described below:

1. Samples across menstrual cycle phases (proliferative, secretory, and menstruation) into one category, and those on hormonal medication into another.
2. All disease stages are grouped into one category; that is, we do not assess disease stage-specific DGE.
3. We assess DGE for the peritoneum and endometrium separately. Consequently, CP, PP, and PeLs create one sample group, while CE, PE, and DiE constitute another sample group.

We carry out the DGE analysis on the following control vs. target categories, each for (combined) menstrual cycle and hormonal medication categories:

1. CE vs. PE.
2. CE vs. DiE (inclusive of all disease sites).
3. CP vs. PP.
4. CE vs. PeL.

### 2.2 GSE51981

Tamaresis et al. (26) compiled genomic data from endometrial samples of 148 women with and without ES, where the control subjects were further subdivided into those with and without common (non-ES) uterine/pelvic pathologies. Their data also included information about the ES disease severity (minimal to mild or moderate to severe) and menstrual cycle phases (proliferative, early, and mid-secretory phases). In our work, we utilize the GSE51981 dataset and perform our DGE analysis by considering all labeled ES as our target samples and controls as those labeled as non-ES, excluding the presence of any uterine/pelvic pathology, and considering all phases of the menstrual cycle. This selection yields a total of 111 samples.

## 3 Methods

We perform our DGE analysis using established R programming language-based packages. We use GEOquery (108) to retrieve the corresponding raw data, along with the associated phenotype and meta-data made available on GEO for both our GSE141549 and GSE51981 datasets. In the case of GSE51981, we additionally use the biomaRt package (https://doi.org/10.18129/B9.bioc.biomaRt) (and the getBM function) to map between ENSEMBL ID information and gene names. We then use DeSeq2 (https://doi.org/10.18129/B9.bioc.DESeq2) to perform the DGE analysis with the “Wald” test type and “local” dispersion fitting, recording the *p*-values, Benjamini–Hochberg method-adjusted *p*-values (*p*adj), and Log_2_[fold change (FC)] for each sequenced gene. Wherever appropriate, we also used “pathview” to help retrieve genes belonging to pathways of interest within our analysis. Specifically, we query genes participating in the NF-κB (NF-κB; kegg: hsa04064), TNF signaling (hereafter TNF, kegg: hsa04668), cytokine–cytokine receptor signaling (hereafter CC; kegg: hsa04060), and histidine synthesis and metabolism (hereafter HSM; kegg: hsa00340) pathways. See the data availability statement for details on the data and code products made available.

## 4 Results

In this section, we discuss the results of our DGE analysis applied to both GSE141549 and GSE51981 datasets.

### 4.1 GSE141549 DGE analysis results

#### 4.1.1 Control vs. eutopic endometrium and patient peritoneum

**Figure 1:**
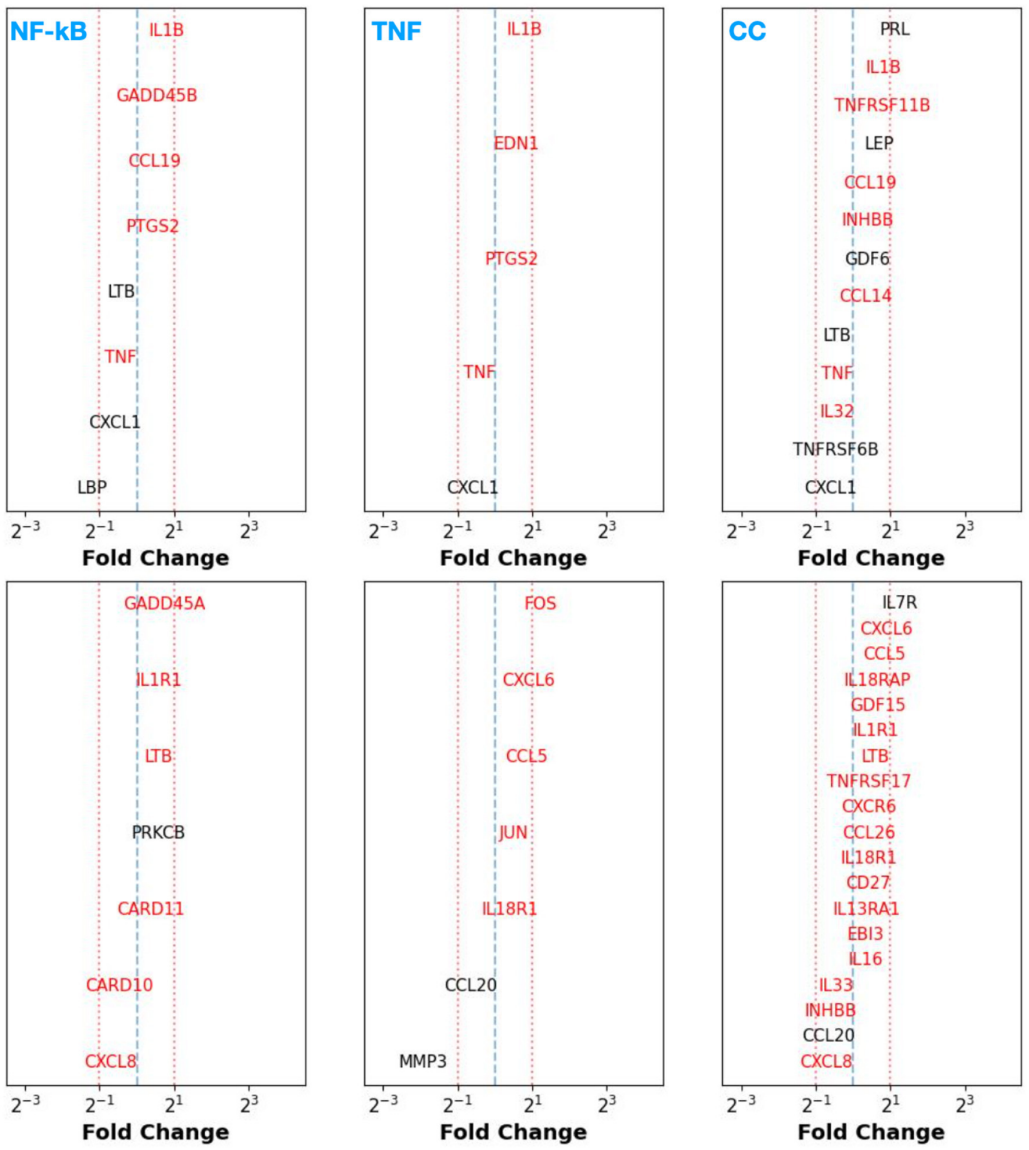
Top row: fold change (FC) of differentially expressed genes [adjusted *p*-value (*p*_adj_) < 0.05 in black] between control endometrium (CE) vs. patient (eutopic) endometrium (PE) samples participating in the NF-κB **(left)**, TNF-signaling **(middle)**, and cytokine–cytokine receptor signaling **(right)** pathways sorted in decreasing order of FC from top to bottom. Genes that have a non-adjusted, native *p*-value < 0.05 are shown in red text. Bottom row: Same as in top row, but for the control peritoneum (CP) vs. patient peritoneum (PP) case.

We assess the differential expression FC for those genes (with *p*-values < 0.05) involved in the NF-κB, TNF, and CC signaling pathways between control endometrium vs. patient eutopic endometrium (CE vs. PE; top row of Figure 1) and control vs. patient peritoneum (CP vs. PP; bottom row of Figure 1).

From this exercise, regardless of whether we consider CE vs. PE or CP vs. PP, we find that there are no specific genes [even when considering the more lenient *p* < 0.05 as opposed to adjusted *p*-value (*p*_adj_) < 0.05] that exhibit significant down or upregulation in the investigated inflammatory pathways. This suggests that the eutopic endometrium and peritoneal tissue (in the absence of any lesions) in patients with endometriosis display no significant genetic changes that contribute toward an enhanced inflammatory response when compared to the disease-free control state.

#### 4.1.2 Control vs. deep infiltrating endometrium and peritoneal lesions

**Figure 2:**
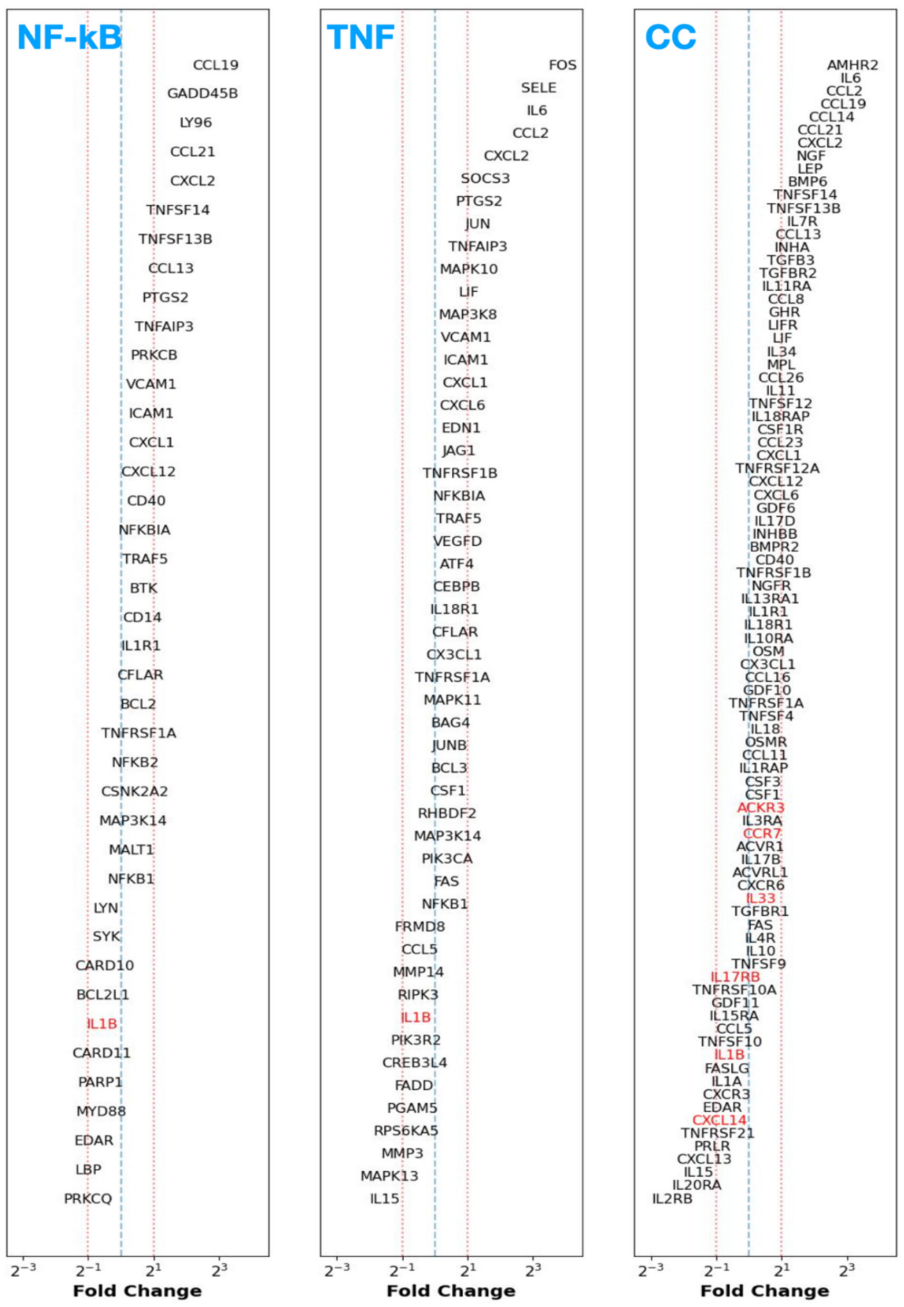
Fold change (FC) of differentially expressed genes [adjusted *p*-value (*p*_adj_) < 0.05 in black] between control endometrium (CE) vs. deep infiltrating endometriosis (DiE) samples participating in the NF-κB **(left)**, TNF-signaling **(middle)**, and cytokine–cytokine receptor signaling **(right)** pathways sorted in decreasing order of FC from top to bottom. Genes that have a non-adjusted, native *p*-value < 0.05 are shown in red text.

Next, we investigated the DGE for the CE vs. DiE case and show the results in Figure 2. It is immediately evident from this figure that various genes in the NF-κB, TNF, and CC pathways exhibit overexpression and underexpression when compared to our previous cases of CE vs. PE or CP vs. PP. We find that genes coding for interleukins (ILs), notably IL-6, exhibit markedly increased expression (~4×), followed by ~2 × expression for IL-11 and IL-34. In contrast, genes coding for IL-15 and IL-19 are ~2.5 × and ~2 × downregulated, respectively. Prostaglandin-endoperoxide synthase 2 [PTGS2, which is also known as cyclooxygenase-2 (COX-2)] shows an enhanced (>2×) expression. We also found a marked elevation (~3×) in IL-7 receptor (IL-7R) expression, followed by a relatively mild elevation in expression (1.5 > FC > 2) among IL1-R1 and IL-18R1. Furthermore, the expression of genes coding for the assembly of IL receptor subunits or accessory proteins, such as IL-11RA and IL-18RAP, exhibits a factor of two overexpression. In contrast, IL-13RA1 and IL-10RA show relatively lower expression (1 < FC < 2). Simultaneously, IL-2RB, IL-20RA show < 2 × downregulation. Furthermore, we also note that various genes coding for the family of chemokine ligands, such as Chemokine (C-C motif) ligand 2 (CCL2), CCL13, CCL14, CCL19, CXCL2, CXCL6, and CXCL12 show more than ~2 × expression.

Concerning the adhesion molecules, we find that vascular and intercellular adhesion factors (VCAM1 and ICAM1, respectively) show ~2 × overexpression, and, notably, Selectin (SELE) shows >4 × overexpression. Regarding the growth factors, we observe that the nerve growth factor (NGF) exhibits a substantial overexpression (~3×) in conjunction with ~1.5 × higher expression of the NGF receptor (NGFR). Vascular endothelial growth factor D (VEGFD) also shows relatively milder increased expression of ~1.5×. Transforming growth factor (TGF) beta 3 (TGF-β3), and TGF beta receptor 2 (TGF-βR2) coding genes show ~2 × overexpression. Finally, genes coding for various tumor necrosis factor (TNF) superfamily proteins (TNFSF), such as TNFSF12, TNFSF13B, and TNFSF14, show elevated expression (>2×), alongside ~1.5–2 × expression on corresponding TNF receptor superfamily (TNFRSF) subunits (e.g., TNFRSF12A and TNFRSF12B). Interestingly, we find that TNFRSF21, an activator of the NF-κB pathway that induces apoptosis, is downregulated by a factor of 2.

**Figure 3:**
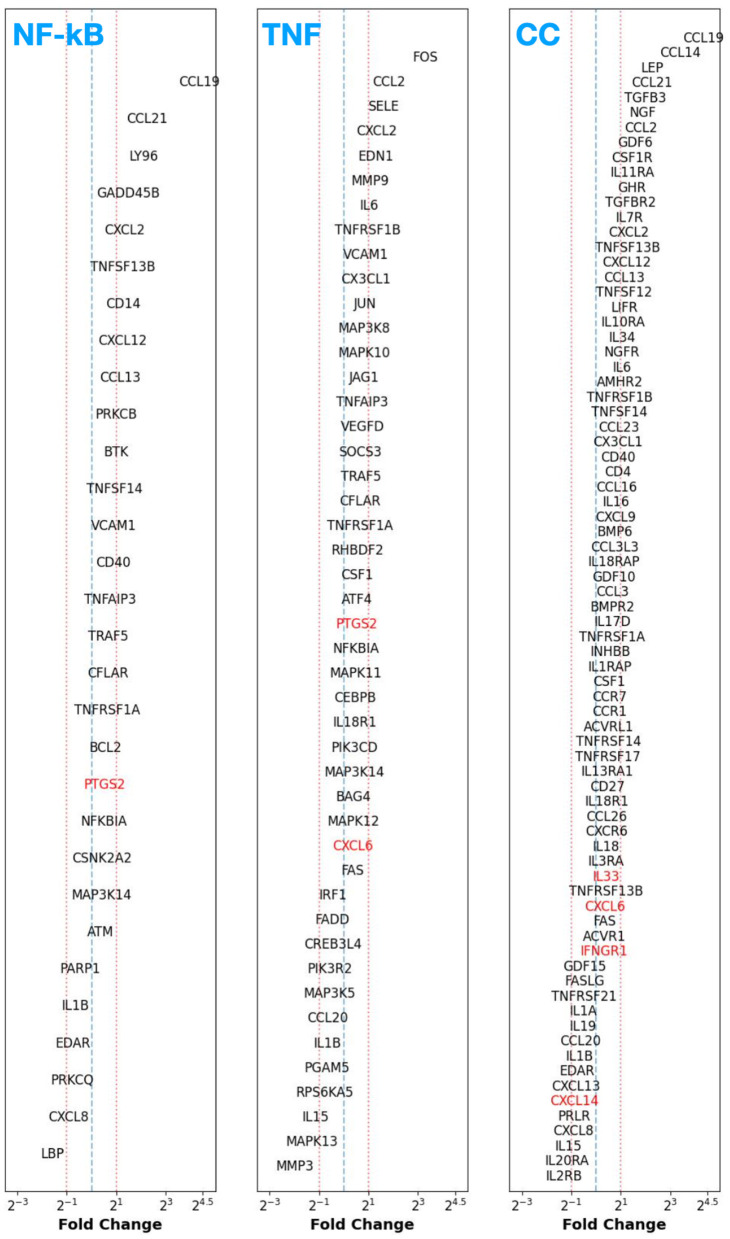
Fold change (FC) of differentially expressed genes [adjusted *p*-value (*p*_adj_) < 0.05 in black] between control endometrium (CE) vs. peritoneal lesions (PeL; see Section 2 for more details) samples participating in the NF-κB **(left)**, TNF-signaling **(middle)**, and cytokine–cytokine receptor signaling **(right)** pathways sorted in decreasing order of FC from top to bottom. Genes that have a non-adjusted, native *p*-value < 0.05 are shown in red text.

In Figure 3, we present the DGE FC results for the case of CE vs. PeL. Compared to the results from CE vs. PE, the CE vs. PeL case demonstrates a more pronounced over- or under-expression across the three pathways (and more notably in the CC signaling). We find that the Genes coding for various CC ligands and corresponding receptor complexes, for example, CCLs 2, 13, 14, 19, 21, and chemokine (C-X-C motif) ligands (CXCLs) 2, 12 show ~2–8 × overexpression, whereas CXCL1s 8 and 13 show ~2 × downregulation. Similarly, we note that IL-6, IL-34, and IL-7R show ~2 × upregulation, followed by IL-16 with ~1.5 × upregulation, and IL-15 shows a ~2 × downregulation. We also find that the genes governing the growth factors (and associated receptors) NGF, NGFR, growth hormone receptor (GHR), TGF-β2, and TGF-β3 show ~>2–4 × upregulation, and adhesion factors VCAM, SELE, and VEGFD show ~1.5–3 × overexpression. Finally, we also note that the TNFSF family of genes (e.g., TNFSFs 12 and 13B) also show ~1.5–2 × upregulation. It is worth highlighting that the observed gene-expression trends in the CE vs. PeL case follow a similar dysregulation pattern to the CE vs. DiE case (see Figure 2), which is expected and also reassuring as PeLs are indeed ectopic endometrial tissue. However, we do find some notable differences between the CE vs. DiE and CE vs. PeL cases. Some of the genes' up- or downregulation is more pronounced in the DiE than in PeL. For example, IL-6, SELE, and CCL2 show more upregulation in DiE than in PeL. At the same time, CCLs 14, 19, and TGF-β3 follow an opposite trend, showing less upregulation in DiE compared to PeL. These observations highlight the upregulation of various key genes that govern inflammation triggering and signaling, immune cell homing and activation, cellular adhesion, and neuroangiogenesis, underscoring the complex role of the immune-mediated inflammatory microenvironment in ectopic (deep) endometrial tissue and peritoneal lesions in patients with ES.

**Figure 4:**
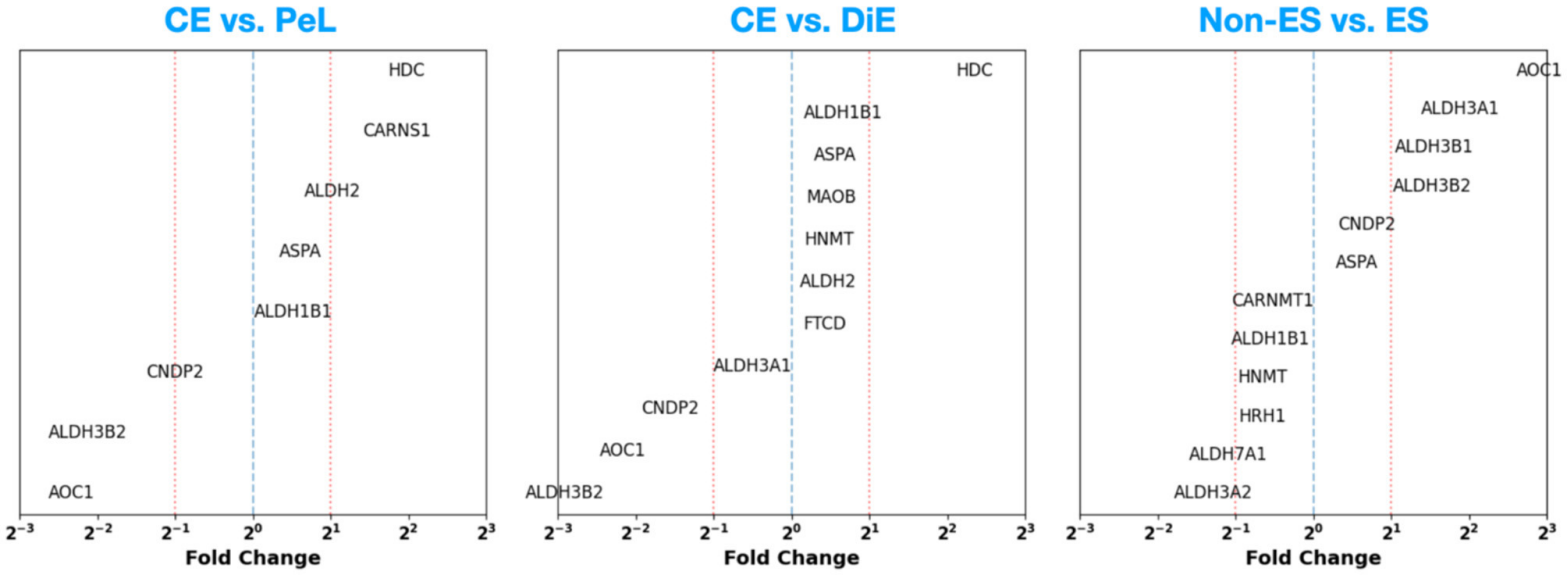
Fold change for genes participating in the histamine synthesis and metabolism pathway based on DGE analysis performed on the GSE141549 (CE vs. PeL and CE vs. DiE cases) and GSE51981 (non-ES vs. ES cases) datasets.

Finally, we also assess the results of DGE analysis explicitly for the genes participating in the HSM pathway. In Figure 4, we explore the DGE FC in both the CE vs. PeL (left panel) and CE vs. DiE cases (middle panel). In the case of CE vs. PeL, we observe a notable ~4 × upregulation of histidine decarboxylase (HDC) and carnosine synthase 1 (CARNS1) genes, which are key players in the HSM pathway, especially in the synthesis of histidine and histamine. Similarly, we find that aldehyde dehydrogenase (ALDH) family members ALDH2 and ALDH1B1, as well as aspartocyclase (ASPA), which helps break down N-acetyl L-aspartic acid (NAA), show ~1.5–2 × upregulation. At the same time, we note that carnosine dipeptidase 2 (CNDP2), amine oxidase copper containing 1 (AOC1) that governs the breakdown of histamine, and ALDH3B2 show substantial ~2–6 × downregulation. In the case of CE vs. DiE, we find a very similar picture to the CE vs. PeL case, where the genes HDC are substantially upregulated (~6×) and AOC1, CNDP2, and ALDH3B2 are ~3–8 × downregulated. These results highlight the dysregulation of HSM pathway genes in ES lesions, which is especially suggestive of promoting local histamine availability through its increased synthesis and decreased breakdown.

### 4.2 GSE51981 DGE analysis results

**Figure 5:**
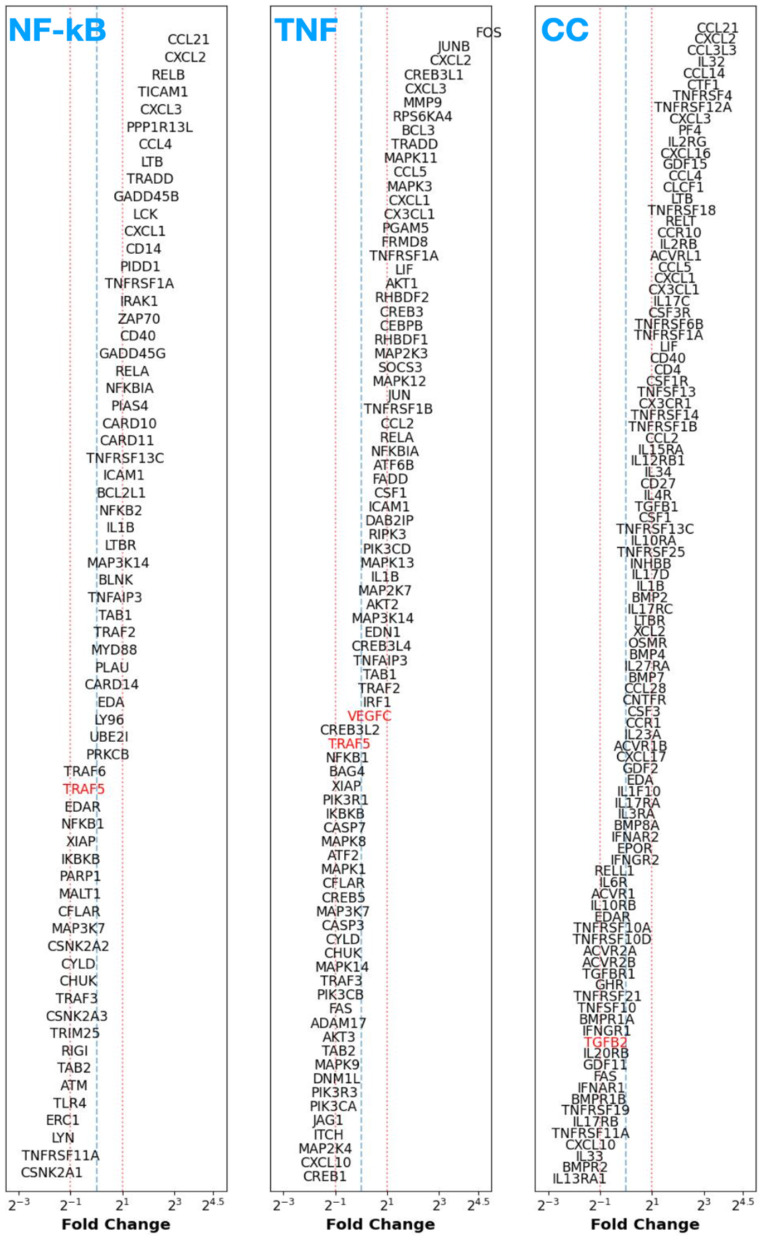
Fold change (FC) of differentially expressed genes (adjusted *p*-value; *p*_adj_ < 0.05 in black) for the non-ES controls vs. ES samples (inclusive of all disease stages, that is, mild to severe) among three pathways: NF-κB **(left)**, TNF signaling **(middle)**, and cytokine–cytokine receptor signaling **(right)**.

In this section, we present the DGE analysis results for the GSE51981 dataset, which contains both non-ES and ES samples. In Figure 5, we present the DGE FC for those genes with *p*_adj_ < 0.05 in the NF-κB, TNF, and CC signaling pathways. We find that many genes coding for proteins participating in the inflammatory and cytokine signaling pathways show substantial differential expression. For example, we find >2 × upregulation in genes coding for ICAM1, ILs 1B, 17C, 32, and 34, various IL receptors and subunits such as IL-4R, IL2-RB and RG, TNFRSFs 1A, 4, 12A, 18, and CC signaling pathways CCL2, CCL4, CCL5, CXCL1, CXCL3, etc. Interestingly, we observe a ~2 × upregulation of the NF-κB inhibitor alpha (NFKBIA), where the encoded protein functions as an inhibitor of NF-κB activation.

Additionally, by assessing the DGE results corresponding to the HSM pathway from the GSE51981 dataset (Figure 4, right panel), we find that AOC1 is ~8 × upregulated. This behavior is contradictory to the CE vs. DiE case in the GSE141549 dataset (where we observed ~4 downregulations). While some ALDH3 family of genes (A1, B1, and B2) are ~2–3 times upregulated, the ALDH7A1 and A2 are ~2 × downregulated. Finally, we also note an interesting result that HRH1 is downregulated by ~1.5–2 ×.

These observations reinforced the analogous interpretations drawn from our GSE141549 results, underscoring the gene-level upregulation of pro-inflammatory signaling cascades in patients with ES. Additionally, our results support the idea that the dysregulated expression of various genes in the ectopic endometrial tissue microenvironment contributes to a simultaneous increase in histamine synthesis and its decreased breakdown.

### 4.3 Impact of using hormonal therapy on DGE in ES

**Figure 6:**
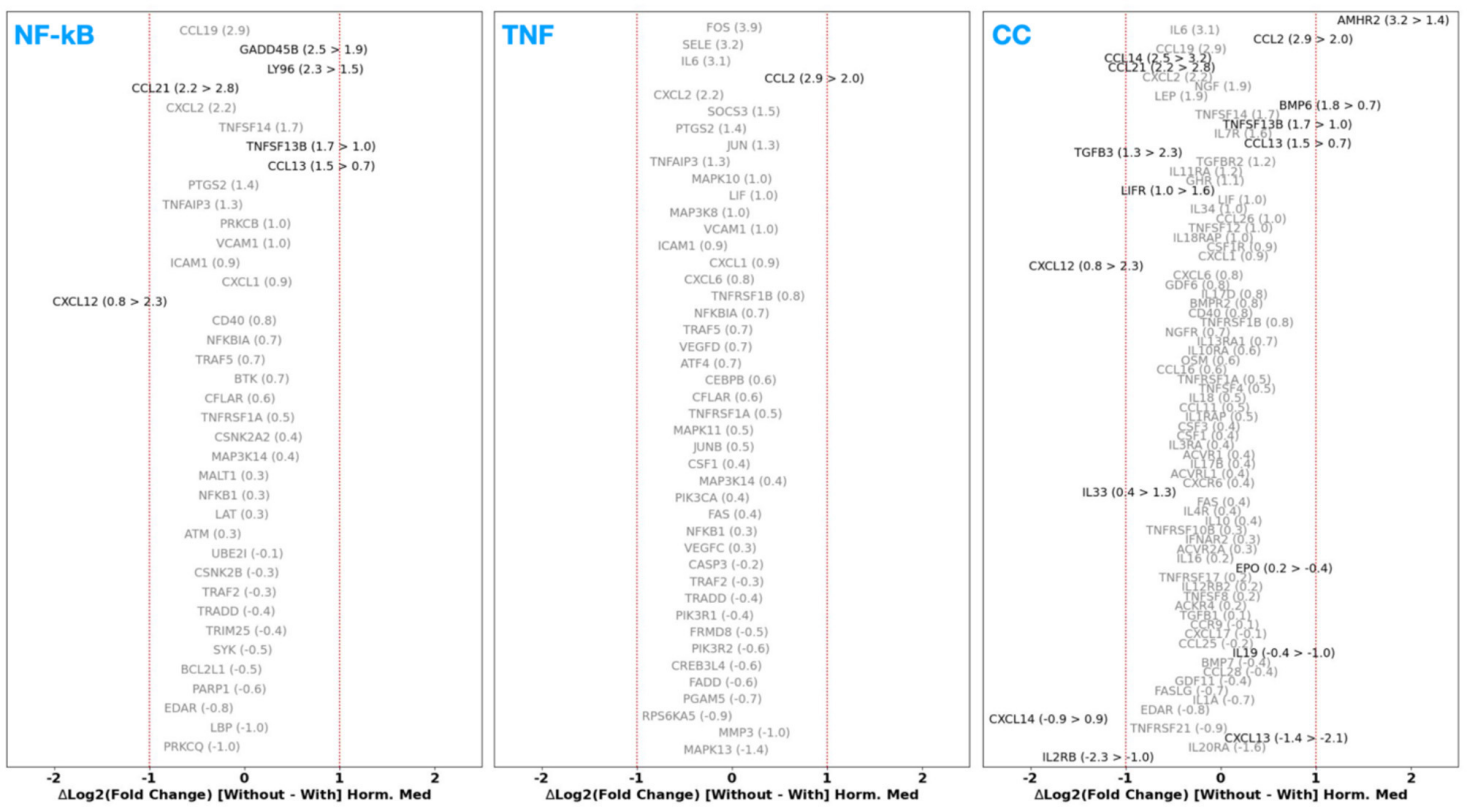
Difference in the differential expression Log_2_ (fold change) of genes within NF-κB, TNF, and CC pathways for the case of control endometrium (CE) vs. deep infiltrating endometriosis (DiE) in patients who are reported to be not using vs. using hormonal therapy. A difference of zero indicates no change in the level of gene up- or downregulation when using (or not) hormonal therapy, whereas a deviation to positive (negative) values indicates relative downregulation (upregulation) when on hormonal therapy.

**Figure 7:**
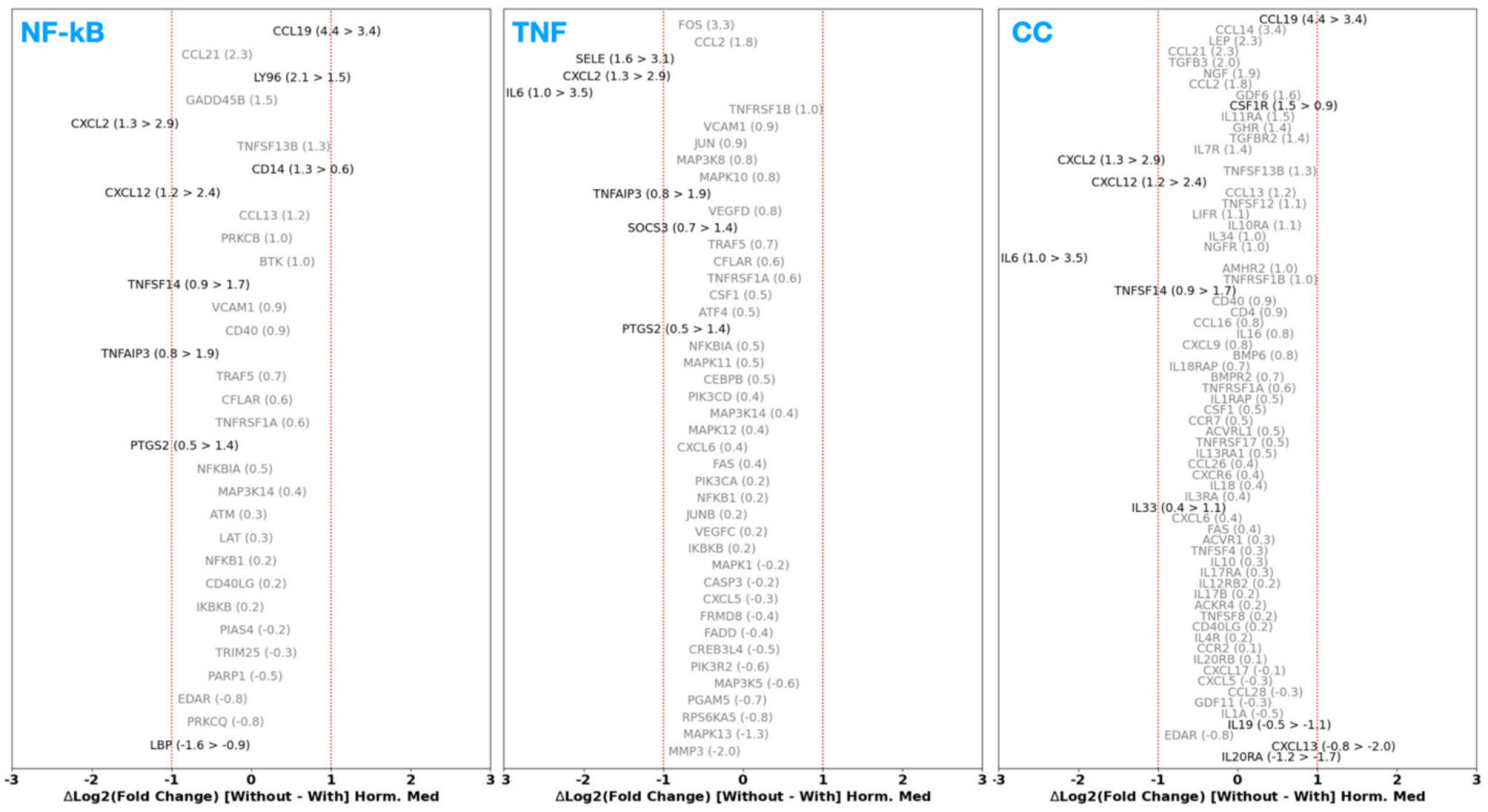
Difference in the differential expression Log_2_ (fold change) of genes within NF-κB, TNF, and CC pathways for the case of control endometrium (CE) vs. peritoneal lesions (PeL) case between those patients that are reported to be not using vs. using hormonal therapy. A difference of zero indicates no change in the level of gene up- or downregulation when using (or not) hormonal therapy, whereas a deviation to positive (negative) values indicates relative downregulation (upregulation) when on hormonal therapy.

**Figure 8:**
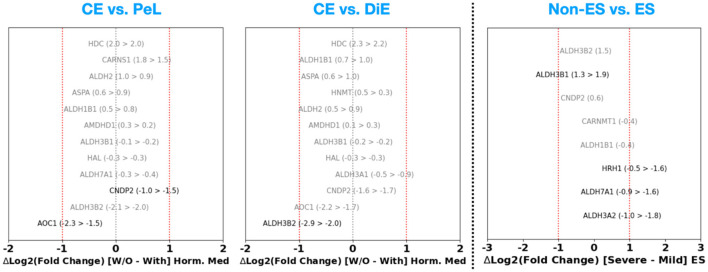
Difference in the Log_2_ (fold change) without and with use of hormonal therapy (in the case of GSE141549) and between moderate-to-severe disease stage vs. minimal-to-mild disease stage (in the case of GSE51981).

Here, we investigate the impact on the DGE among patients with confirmed ES (either DiE or PeL) when using hormonal therapy for ES management. Specifically, for each case of CE vs. DiE and CE vs. PeL, we perform the DGE analysis on two subsets: those who are on hormonal therapy and those who are not, and assess the difference in the FC between the genes commonly up- or downregulated among them. In Figures 6, 7, we present the Log_2_(FC)_w/o_horm−_Log_2_(FC)_horm_ for CE vs. DiE and CE vs. PeL cases, respectively. In Figure 8, we show the Log_2_(FC)_w/o_horm−_Log2(FC)_horm_ for the HSM pathway.

In both cases of CE vs. DiE and CE vs. PeL, we find that the bulk of genes (and their differential expression), belonging to the NF-κB, TNF, CC, and HSM pathways, remained unchanged when on hormonal therapy. However, it is worth pointing out that some genes coding for IL-19, TNFSF13, CCL2, CCL13, CXCL13 have preferentially depressed expression (by ~1.5×), whereas IL-33 and IL-2RB, TGFB3, CCLs (12, 14, 19, 21), and CXCLs (12, 14) show an amplification from ~1.5 × to ~4 × when using hormonal therapy. Furthermore, in the case of PeL, it is worth noting that IL-6, COX-2, and SELE exhibit a 2–3 × amplification in their upregulation when hormonal therapy is employed.

The above insights suggest that hormonal therapy does not directly contribute to suppressing the dysregulated pro-inflammatory or HSM pathways. In fact, in some cases, we find instances of further amplification or upregulation of specific pro-inflammatory signaling pathways.

### 4.4 Variations in DGE as a function of disease severity

In this section, we present the DGE analysis results for the GSE51981 dataset, which contains both non-ES and ES samples, and assess the impact of disease stage (minimal-to-mild ES vs. moderate-to-severe ES) on the regulation of inflammation and histamine mediation genes.

Assessing the up- or downregulation of genes participating in the HSM pathway (Figure 8, right panel), we find that HRH1, ALDH7A1, and ALDH3A2 are more downregulated [ΔLog_2_(FC) ~ 1] in the case of minimal-to-mild disease compared to severe cases. This, especially in the case of HRH1, may be a consequence of the overall upregulation of various inflammatory cascades as shown in Figure 7, and the cellular mechanisms responding to it by counteracting this via downregulation.

**Figure 9:**
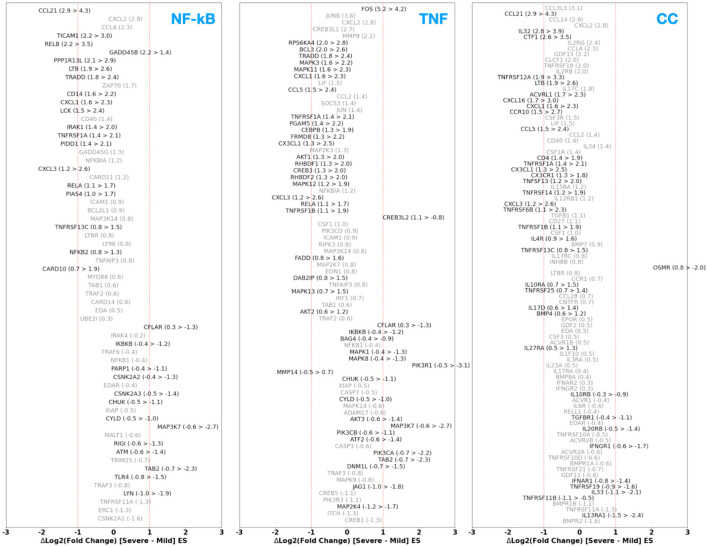
Difference in the differential expression Log_2_ (fold change) of genes within the NF-κB, TNF, and CC pathways for the non-ES vs. ES case between those patients that are reported to have minimal-to-mild ES vs. moderate-to-severe ES.

In Figure 9, we present a comparison of the DGE FC between patients with moderate-to-severe ES vs. minimal-to-mild ES for the NF-κB, TNF, and CC pathways. Firstly, there are a considerable number of genes coding for CAMs, ILs, TNFSFs, and CC ligands that do not show substantial change in their up- or downregulation (i.e., within a factor of 1.5). Next, we observe an interesting trend where certain genes that are, in general, up- or downregulated tend to be more upregulated in the case of minimal-to-moderate ES than moderate-to-severe ES. For example, CCLs 5 and 21; CXCLs 1 and 16; IL-32, IL-17D, and IL-4R; and TNFSF13, TNFRSF 12A and TNFRSF14 all demonstrate a ΔLog_2_(FC) ~ 1. Along the same lines, for example, TGBR1, IL-33, IL-10RB, and IL-20RB, Interferon Alpha/Gamma Receptors Type 1 (IFNAR1 and IFNGR1) showcase more downregulation [up to a factor of 1.5–2; ΔLog_2_(FC) ~ −1] in the minimal-to-mild case compared to the moderate-to-severe case. Nevertheless, there are a few exceptions to this trend, for example, those that are involved in cell growth cycle modulation, such as FBJ murine osteosarcoma viral oncogene homolog (FOS); FOS protooncogene, GADD45A/B, an environmental-stress induced cell-cycle arrester, and Oncostatin M Receptor (OSMR) involved in IL-31 signal transduction, are more upregulated in moderate-to-severe ES cases.

In summary, we find that several genes participating in the pro-inflammatory and histaminic pathways tend to show amplified up- or downregulation in the minimal-to-mild disease stages compared to the moderate-to-severe cases. We interpret this trend as a likely reflection of distinct pathogenesis factors that specifically dominate during the milder disease stages and undergo normalization or compensation as the disease severity progresses. Our findings and interpretations align with those reports from various studies. For example, immune-related gene expression studies by Vallve-Juanico et al. (27), Chen et al. (107), Zygula et al. (28) indicate a more pronounced inflammatory environment in early ES as compared to late stages through increased synthesis of cytokines (28) and more pronounced activation and homing of multiple immune cell types (107). From a general gene expression perspective, a recent study by Cook et al. (29) has also found a trend where several genes showed enhanced dysregulation in milder stages of ES disease. This picture aligns with findings from other transcriptomic studies (26) where genes specifically participating in pro-inflammatory pathways (such as those probed in our work) are notably enhanced in milder stages of ES disease.

## 5 Discussion and comparisons with the literature

In this study, we presented results from a DGE analysis performed on two datasets, specifically focusing on the inflammatory signaling pathways—NF-κB, TNF, CC, and histamine synthesis and metabolism. In this section, we evaluate our results in the context of previous studies in the literature and discuss our interpretations within this framework. Next, we discuss various results from the literature that quantified the impact of H1-antihistamines (H1-As) on the cytokine profiles and pro-inflammatory signaling pathways.

A plethora of recent literature reviews [(11, 30) and the references within] support the complex role played by the immune system in the pathogenesis of ES, especially via the promotion of inflammation on both systemic and microenvironment through cytokine-mediated signaling, vascular and neuroremodeling, and subsequent survival of ectopic endometrial implants through local hormone-mediated growth.

Various cell lines, including macrophages, neutrophils, natural killer (NK) cells, T-lymphocytes, mast cells, and B-lymphocytes, are expected to be present in the normal endometrium, with varying incidences at different phases of the menstrual cycle. A dysregulation of their incidence has been noted in various studies of the endometrium within ES patients. For example, ES patients have been reported to have elevated macrophage counts, especially the M1 subtypes that promote inflammation through cytokine production (31), and peritoneal macrophages have been demonstrated to have NF-κB pathway activation (14, 32). Furthermore, studies (33, 34) have found that degranulated mast cells have been found in the ES lesions, especially in the DiE, hinting at the potentiality and mediation of immune hypersensitivity through release of mast-cell granular contents such as histamine, heparin, and matrix-degradation products.

### 5.1 Cytokines and chemokines

ES has been associated with the presence of impaired or overproduction of various cytokines (e.g., IL-1β, IL-6, IL-8, and IL-10) (35). This, in conjunction with the capacity for the ectopic tissue to release such cytokines, which in turn can activate other pathway factors, can potentiate a persistent inflammatory state in ES (36).

IL-1β is involved in the synthesis of prostaglandins (PGs, e.g., PGE2) through activation of COX-2 and in the recruitment of macrophages and neurogenesis through CCL5 regulation (37, 38). Furthermore, CCL2 and CCL5 serve as attractants for recruited macrophages, thereby yielding an overexpression of COX-2 in ES patients (39). IL-6 is another major cytokine that has been found at elevated concentrations both systemically and in peritoneal fluid of ES patients, where it is secreted by both migrated macrophages in the endometriosis lesion environment and (to a minor part) the endometrial cells (40, 41). IL-6 overexpression and secretion have been linked to endometrial lesion growth (42), and its high concentrations have been correlated with the severity of ES disease (43). Furthermore, IL-6 also contributes to the survival of ES lesions by suppressing the cytotoxicity of NK cells, which can induce apoptosis under normal regulatory conditions (41). Consistent with this literature, our work reveals that IL-6 is overexpressed (~8×) in ES patients regardless of hormonal medication use.

Various other cytokines, such as IL-15, IL-32, IL-33, and IL-34, have been studied in the context of ES [see review by Machairiotis et al. (44)]. IL-15 is a stimulant of T-lymphocyte maturation and plays a role in NK-cell growth, with some reports also indicating angiogenic properties (45, 46). Bellelis et al. (47) studied the concentrations of various cytokines, including IL-15, and found significantly elevated concentrations in the ectopic endometrial tissue of patients with DiE. Our findings from a gene regulation perspective indicate that IL-15 is substantially downregulated. Although our findings align with those of the study by Malvezzi et al. (48), their results differ from those of other studies that have found an increased IL-15 expression (49). We believe that this contradictory scenario is likely a reflection of complex feedback mechanisms at play, governed by estrogen-dependent downregulation of IL-15 gene expression (50).

IL-32 and IL-33 play a crucial role in modulating immune response, where the former is an inducer of other cytokines (e.g., IL-1β and IL-6) and the latter is a modulator of tissue repair (44). Elevated concentrations of both IL-32 and IL-33 have been detected in the peritoneal fluid and ectopic lesions of ES patients (51, 52). In our work, we found an increased expression of IL-32 (~4×) and a decreased expression of IL-33 (~2–2.5×). This suggests that IL-32 sustains a pro-inflammatory environment overexpression, while tissue repair mechanisms are dampened by lowering of IL-33's expression.

IL-34 has been functionally attributed to various critical aspects, including apoptosis, immune regulation, and angiogenesis. Lin et al. (53) studied the role of IL-34 (and other factors, such as VEGF), notably using one of the datasets we utilized in our work (GSE51981), and found an upregulation of IL-34 that is consistent with our findings. Furthermore, in our work, we also see a similar (~2×) upregulation of IL-34 in the case of DiE and PeL.

The cytokine signaling and NF-κB pathway can induce a synergistic positive feedback activation of the TNF signaling pathway, resulting in downstream reinforcement of the inflammatory processes. Macrophages within the pelvic endometriotic lesions have been shown to produce excess TNF-alpha (as evidenced by greater levels in peritoneal fluid in ES patients) (15, 54) in response to NF-κB activation via COX-2 overexpression (8, 55), which in turn can participate in further synthesis of PGE2 and cytokines such as IL-6 and chemokines such as CCL2 (56).

In addition to their inflammatory effects, the aforementioned cytokines and chemoattractants can have cascading effects on the steroid-dependent pathways (progesterone P4 and estradiol E2), which in turn govern the growth of endometrial lesions [see Figure 1 in García-Gómez et al. (57)]. For example, the overexpression of COX-2 and subsequent PGE2 synthesis lead to an increased level of E2, which can in turn induce cytokine and prostaglandin synthesis (42), through the overexpression of aromatase (58). Increased levels of E2 can help sustain ES lesion survival by impairing the apoptosis mechanism regulated by the TNF pathway (59).

### 5.2 Cellular adhesion, vascular- and neurogenesis factors

Impairment or overexpression of Intercellular and Vascular Adhesion Molecules (e.g., ICAM1 and VCAM1, respectively) is predominantly associated with their essential role in the migration of leukocytes and lymphocytes toward a target/inflammation location (60, 61). Several studies (17–19, 62) have indicated the impairment of ICAM1 and VCAM1 (specifically overexpression) in ectopic ES lesions as well as higher systemic concentrations of their soluble versions in ES patients. This picture is consistent with our findings, specifically the overexpression of ICAM1 and VCAM1, particularly in deep infiltrating endometriosis tissue and in peritoneal lesions (PeL) or eutopic endometrium (PE) of ES patients.

Various studies have explored the differential expression of vascular and neurogrowth factors such as Vascular Endothelial Growth Factors (VEGFs), NGF, and TGF-β within ES patients. Szubert et al. (20) found no significant differential expression of VEGF, but a downregulation of NGF in eutopic endometrial tissue of ES patients. In our work, we did not find VEGF to be differentially expressed in either eutopic or ectopic endometrial tissues. However, we did find NGF to be overexpressed (~4×) in the case of ectopic tissue in patients with DiE. Makabe et al. (63) studied the impact of synthetic progestin and estradiol (specifically Drospirenone) on regulating NGF (and other cytokine) expression and found that such a combined treatment shows substantial downregulation of NGF when compared to controls. We find a contrasting result, where no significant difference in NGF differential expression with and without hormonal medication use. This may be because the study by Makabe et al. (63) focused on a specific drug of choice, and our results are based on non-specific and potentially much broader choices of hormonal drugs used. Sikora et al. (64) investigated the concentrations of TGF-β (and its isoforms TGF-β1, β2, and β3), which play a wide role in various immunomodulatory functions [see Figure 1 in Sikora et al. (64)]. They found it to be substantially dysregulated (with increased concentrations) in the peritoneal fluid of ES patients, which could favor the formation of ES lesions. This is consistent with our findings of ~2 × upregulation of TGF-β1 and TGF-β2 genes, which did not change in the case of hormonal therapy usage or with the disease severity (mild vs. severe). Furthermore, it is worth noting that various interleukins discussed in the previous section (e.g., IL-15, IL-34, etc.) also exhibit synergistic angiogenic properties that can, in turn, stimulate VEGF and other growth factors.

### 5.3 Oncogenic factors

Some researchers have studied ES in the context of endometrial cancer (EC) and have identified various genes whose differential expression and mutations have been associated with an increased sensitivity to EC. Gowkielewicz et al. (37) identified that the Anti-Mullerian Hormone Type 2 Receptor (AMHR2) is overexpressed in EC tissue of nearly all patients. Interestingly, in our DGE analysis of the CE vs. DiE samples, we found a nearly 8 × upregulation of AMHR2 in the case of non-hormonal therapy and a moderate ~2.6 × in the case of patients on hormonal therapy. This suggests that preoncotic, genetic-level changes are at play in DiE that are also common to EC. Notably, hormonal therapy seems to substantially dampen this AMHR2 overexpression. Similarly, studies (65–67) have associated mutations and differential expression of PIK3CA and PIK3R1 (which govern apoptosis) in both ES lesions and EC tissues (68, 69). Upregulation of PIK3CA and PIK3R1 has been associated with inhibition of cellular apoptosis and, as such, tumorigenesis (70). In our analysis, we find that PIK3CA and PIK3R1 show a stronger downregulation (~8 × and ~4 ×, respectively) in the minimal-to-mild ES disease stage than in the moderate-to-severe ES disease stage (downregulation of ~1.5×). This indicates that as ES progresses in its severity, the relative dysregulation of PIK3CA and PIK3R1 likely tends to become more upregulated in nature.

It is worth highlighting that Mirza et al. (71) performed a DGE analysis using various datasets, including GSE51981 (used in this work), but in a much broader context and not limited to the four specific pathways that we analyzed. Importantly, we also find a consistent upregulation in the cases of both GSE141549 and GSE51981 datasets when compared to Log_2_(FC) by Mirza and Abdel-Dayem (71); for example, protooncogenes FOS, JUN/JUNB (jun class). An overexpression of FOS (as well as JUN) is associated with impaired endometrial cell proliferation and endometrial oncogenicity (72). Furthermore, we find that FOS is comparatively more upregulated in the moderate-to-severe disease stage (see Figure 7), and that its upregulation is only minimally impacted by the use of hormonal therapy for both peritoneal lesions and deep infiltrating endometriosis (see Figures 4, 5). As such, our results presented in this study support and reinforce the notion of common oncogenic pathways between ES and EC.

### 5.4 Histidine metabolism and associated genes

Histidine, through histidine decarboxylase (HDC), is metabolized to histamine, which exhibits a broad range of biological functions [see reviews by Stojković et al. (21) and Mahdy and Webster, (73)]. Histamine is metabolized through two major pathways, governed by the enzymes histamine N-methyltransferase (HNMT) and diamine oxidase (also commonly referred to as amine oxidase copper-containing 1; AOC1). Mast cells and macrophages, potentiated by stimuli from other cells, can synthesize and release histamine (along with other cytokines and chemokines) to cause downstream pro-inflammatory cascades (74, 75).

While there is a plethora of literature that studied and uncovered the role of histamine and it's mediated pathways in various cancer pathogenesis (e.g., breast and colon cancers, melanomas, etc.), genes participating and modulating the histidine- (and thereby histamine-) mediated pathways in the context of ES have been very sparsely explored. Fan et al. (76) studied the role of metabolism-related gene expression in EC patients and found that the HNMT gene was downregulated compared to the control population. Jeda et al. (77) assessed the role of histaminergic pathways and participant genes and found HNMT to be differentially expressed (>1.5 × FC) in grade-2 EC. Wang et al. (78) quantified the expression of all four types of histamine receptors (HRH1–4) along with the response of EC cells to histamine presence. They find that HRH1 and HRH2 are preferentially expressed in EC cells and that histamine-HRH1 communication plays a dominant role in the upregulation of Aldehyde Dehydrogenase 1 (ALDH1). Interestingly, they also found that the action of histamine promotes cellular migration, evades apoptosis, and supports resistance to chemotherapeutic agents, which is countered by H1 antagonists (i.e., H1 antihistamines). In our work, we find that HRH1 expression is downregulated (~1.75×) in ES patients, and this downregulation is more pronounced in milder disease (~-3×) than in severe disease (−1.5×).

Increased expression of the ALDH1A family and its isoforms (A1, A2, and A3) have been associated with poorer prognosis and invasiveness in EC (and also in other cancers) as their prevalence is reported in the so-called “cancer-initiating-cells” (79), Interestingly, Ma et al. (80) showed increased expression of all three isoforms in patients within ES.

Furthermore, Aldehyde Dehydrogenase 2 (ALDH2) enzyme can counteract reactive aldehydes, and studies, such as McAllister et al. (81), have demonstrated a reduced ALDH2 activity owing to downregulated ALDH2 gene expression in ectopic endometrial tissue of ES patients. Furthermore, Hellenthal et al. (82), in their study, have demonstrated that an increased expression or activation of ALDH2 can yield beneficial outcomes in ES (e.g., regression of lesions). In our study, we find an expression of ALDH2 (~1.5–2×) in the case of both DiE and PeL, likely indicating ongoing mitigation pathways counteracting the disease progression. Cui et al. (83) did a comprehensive quantitative gene expression study of all ALDH genes (1–19) amongst various cancers and found that ALDH1B1 and ALDH3A2 showed increased expression in EC, whereas ALDH2 showed a decreased expression. In our work, we find that ALDH1B1 is moderately upregulated (~1.5×) in the case of DiE and PeL, and ALDH3A2 is ~2 × downregulated in ES patients.

Several key points from the literature, alongside our results, made in this subsection highlight the dysregulation in the histamine-dependent pathway as well as underscore the common pro-oncogenic factors between ES and EC. Furthermore, this discussion also highlights the strong need for more comprehensive quantification of histamine's impact on ES progression.

## 6 Summative conclusion of our DGE analysis

Thus far, we have presented results of differential gene expression (DGE) analysis performed on two large genomic datasets and discussed them in the context of results by various other works. We focused on assessing the up- or downregulation of genes involved in three pathways that participate in inflammation cascades (NF-κB, TNF, and cytokine–cytokine signaling) and histamine synthesis and metabolism. As permitted by the used dataset, we present and interpret our DGE analysis results: for various lesion/tissue types [patient peritoneum (PP), patient endometrium (PE), peritoneal lesions (PeL), deep infiltrating endometriosis (DiE)], when using or not using hormonal therapy, as a function of disease severity (minimal to mild; moderate to severe). We present a concise summary of our analysis below.

1. Overall, consistent with the emerging picture from various other studies, our analysis revealed a dysregulated expression of various genes among ES patients. Some notable mentions are those involved in pro-inflammatory signaling: ILs (6, 15, 32, 33, and 34), COX-2, CCLs (2, 4, and 5), TNF-alpha (and TNF superfamily members, e.g., TNFSFs 12 and 14); Cellular adhesion (ICAM1, VCAM1, and SELE); neuroangiogenesis (VEGF, NGF, and TGF-β).
2. We find that the above-mentioned dysregulated expression is more notable and pronounced in the case of PeL and DiE tissue compared to the minimal changes in PP and PE of ES patients. This implies that eutopic endometrium and lesion-free peritoneal tissue in ES patients do not exhibit significant pro-inflammatory genetic dysregulation compared to a disease-free control state.
3. We observe notable dysregulation of genes participating in the histamine synthesis and metabolism pathway in both DiE and PeL cases. Our findings indicate a scenario where the ectopic endometrial tissue microenvironment might be supporting an increased histamine synthesis (through an upregulation of HDC) and local availability (as indicated by a feedback HRH1 downregulation) via its reduced breakdown (e.g., through downregulation of AOC1 and CNDP2), and a reduced potential to clear reactive aldehyde species (via a downregulation of ALDH family members).
4. By assessing the differences in gene regulation among ES patients with and without hormonal therapy (in the cases of PeL and DiE), we find that hormonal therapy overall had a minimal impact on altering the (dys)regulation of the majority of genes participating in pro-inflammatory cytokine signaling and the histaminergic pathways. While it is worth noting some instances where it downregulates genes that have downstream inflammatory actions (e.g., IL-19 and CCL2), we also find cases of relative upregulation of genes that have downstream inflammatory or tissue-damaging actions (e.g., increased upregulation of IL-6, COX-2, IL-33, and TGF-β).
5. Assessing the differences in gene regulation as a function of disease severity, while there were a substantial number of genes that did not have a significant differences, we find that specific genes involved in inflammation (e.g., IL-32, IL-33, and CCL5) and histamine cascades (e.g., HRH1, ALDH3, and ALDH7 family) showed an amplified dysregulation (i.e., more up- or downregulation) in minimal-to-mild disease stage as compared to a moderate-to-severe case.
6. Additionally, we found that various apoptosis-modulating genes exhibit substantial dysregulation, including FOS protooncogene, GADD45A/B, AMHR2, PIK3CA, and PIK3R1, as well as the JUN family. Their dysregulation is relatively more upregulated in severe disease stage, but substantially less dysregulated when using hormonal therapy (e.g., for FOS and AMHR2).
7. Considering the additional context from studies that reported the dysregulation of these genes in both ES and EC, our findings support the notion of common pathways between ES and EC, while also highlighting the potential pro-oncogenic changes within a sustained pro-inflammatory ES environment.

## 7 Broader perspective from a histamine and antihistamine context

In this section, we further our discussion by expanding the aforementioned summative points from our DGE analysis into a much broader context, involving the role of mast cells and histamine in mediating inflammatory cascades, as well as the anti-inflammatory and mast-cell-stabilizing effects of H1-antihistamines (H1-As). By placing these broader points in the context of ES and existing therapeutic options, we provide key interpretation-based insights that lend support toward considering H1-As as a potential therapeutic option for ES and its broader implications.

### 7.1 Histamine, H1-antihistamines, and their impact on cytokine profiles

Nearly a century of research since its discovery, histamine's role in governing various vital biological functions (e.g., neurotransmission, modulation of immune cells and inflammation, and gastric acid secretion) has become evident; it has only been expanding with more recent experiments. Such functions are made possible through the histamine's binding to the histamine receptor(s) HRH1, HRH2, HRH3, and HRH4, where the expression of these receptors varies widely depending on the cells in question. From an immunological cell-line standpoint, histamine is predominantly secreted by mast cells (and basophils) and binding to histamine receptor type 1 (HRH1) has been attributed to the governance of various downstream signaling cascades (73, 84).

The role of granulocyte-mediated histamine release and its action has been attributed to various diseases, not only in predominantly discussed and highlighted allergic conditions, such as asthma, atopic dermatitis, and chronic urticaria, but also multiple sclerosis and rheumatoid arthritis [see references within Stojković et al. (21)]. Patients with the aforementioned conditions have been shown to have both local and systemic high histamine levels. Furthermore, the histamine-HRH1 interaction has been reported as an inducer of pro-inflammatory cytokine secretion (e.g., IL-1, IL-6, and IL-8) and upregulation in the expression of adhesion molecules (e.g., ICAM-1 and VCAM-1), and an activator of the NF-κB pathway (85, 86).

Mast cells have also been increasingly associated with endometriosis. Various studies have found the prevalence of activated and degranulated mast cells within both peritoneal fluid and lesions of endometriosis patients that are diffusely infiltrated (33, 34, 87, 88). Zhu et al. (89) found that estrogen-dependent stimulation can lead to the activation of mast cells, resulting in downstream effects. Li et al. (90) found that mast-cell granules help potentiate angiogenesis, and Anaf et al. (34) found evidence that degranulated mast cells potentiate neurostimulation and neuropathic pain (supplemented by NGF) in the case of ES lesions (more specifically in DiE). As a result of this increasing evidence for dysregulated mast-cell behavior within ES patients, various studies have suggested mast-cell stabilizers (e.g., ketotifen) as a potential therapeutic avenue (91–93, 105).

H1-As are a class of drugs that function by stabilizing the HRH1 receptor through inverse agonist behavior (84), which are widely available and have been used for a plethora of conditions ranging from nausea to chronic urticaria and allergic rhinitis. Functionally speaking, H1-As can be categorized into Generation 1 and Generation 2 [hereafter referred to as G1 and G2, respectively; also Simons and Simons (94)]. The primary distinction between G1 and G2 H1-As is the former's ability to cross the blood–brain barrier and impart its effect on the CNS (where histamine serves as an important neurotransmitter). It is the reason for its sedative effects. G2 H1-As, on the contrary, does not cross the blood–brain barrier and as such has minimal (if any) central nervous system (CNS) effects. Furthermore, G1 H1-As also have a relatively lower affinity toward HRH1 and, as such, have been associated with acting on cardiac ion channels and potentiating side effects such as QT prolongation and malignant arrhythmias. G2 H1-As, although theoretically carrying the same risk, safety profile studies have shown that such side effects (not just cardiac, but in general) are pretty rare.

Several studies have investigated the local and systemic anti-inflammatory effects of H1-As in various allergic diseases. Notably, H1-As (e.g., Desloratidine and Levocetirizine; both G2 H1-As) have been demonstrated to suppress the secretion of various pro-inflammatory cytokines (22) such as IL-2, IL-6, TNF-alpha, and IL-31; and chemokines CCL5 and ICAM-1 [e.g., Giustizieri et al. (95)]. Alongside such anti-inflammatory properties, many studies have also uncovered H1-As mast-cell stabilization properties. For example, Levi-Schaffer et al. (96) have found that Desloratidine and Fujimura et al. (23) have found that Desloratidine and Cetirizine (also a G2 H1-As) have demonstrated mast-cell stabilization.

### 7.2 Existing and on-the-horizon therapeutic avenues for ES

Various management strategies (both surgical and non-surgical) are established for the management of endometriosis [see the comprehensive pharmacological review by Mitronovici et al. (11)]. Non-surgical options involve predominantly the use of hormonal therapy, which in itself has a spectrum of options. Broadly speaking, all these drugs target the estrogen synthesis pathway with the goal of suppressing the estrogen-mediated growth and proliferation of ectopic endometrial lesions. Oral combined estrogen and progesterone pills or progesterone-only (often referred to as “mini” pills), periodic progesterone-only injections, and sustained-release progestin-releasing implants (e.g., etonogestrel and levonorgestrel) are commonly used options. Additionally, aromatase inhibitors, GnRH agonists, and antagonists that act on the pituitary-hypothalamic-ovarian axis for estrogen suppression have also been successfully used in cases where the classical regimen fails. However, it is important to note that short- and long-term use of the aforementioned estrogen modulation drugs come with a plethora of side effects such as abnormal vaginal bleeding, hot-flashes and headaches, weight gain and fluid retention, mood changes and depression, hypercoagulability, bone density loss and increased risk of osteoporosis [e.g., see table 1 in Mitranovici et al. (11)].

Furthermore, ongoing investigations into using anti-angiogenic drugs, selective estrogen, progesterone, and aromatase receptor modulators are underway. Selective TNF-alpha and tyrosine kinase inhibitors, as well as NF-κB modulators, are also being considered as novel and promising therapeutic targets for ES, specifically targeting inflammatory pathways. However, comprehensive studies on their efficacy and safety profile are yet to be fully understood.

### 7.3 Reflections and interpretations: synergistic use of H1-As in ES management?

Taking into consideration the gene-expression analysis results, synergistically with various discussion points from the literature above, the following set of broad, distilled observations emerges:

1. Inflammation is a key factor in ES, driven by the amplified expression and synthesis of pro-inflammatory ILs, chemokines, cellular-adhesion molecules, growth factors, and immune cell attractants and recruiters.
2. There are oncogenic/tumorigenic factors that demonstrate amplified expression in both ES and EC.
3. Histamine synthesis, its mast-cell mediated release, and downstream cascades (cytokine and chemokine regulation) potentiated through histamine-receptors (e.g., HRH1) are dysregulated in ES and a plethora of cancers (including EC, breast cancer, etc.).
4. H1-Antihistamines' anti-inflammatory and mast-cell-stabilizing role has emerged, whereby both the release of histamines (as well as other granular contents) and the ongoing action of histamines in stimulating pro-inflammatory cascades are countered.

This perspective opens an interesting interpretation and key thought avenue—“Can H1-As be used in the context of ES?”; As a consequence, several related questions also emerge:

1. Can the systematically reduced cytokine profile through H1-As aid in halting or even reversing the ES lesion growth (potentially giving space for immune clearance)?
2. Can the use of H1-As within ES and its downstream effects improve the overall pain burden (by reducing hyperalgesia) and quality of life?
3. Can H1-As aid in a more substantial synergistic effect (with hormonal therapy) in suppressing the amplification of oncogenic pathways?
4. How does the use of H1-As impact the progression of ES disease severity?

However, to the best of our knowledge, studies to date have not systematically explored the role of H1-As in the context of ES therapeutics, and the aforementioned questions remain unanswered. Lending support to our line-of-thought and recommendation, H1-As indeed have been discussed in a more second-order context in ES, for example, Anaf et al. (34) speaks of mast-cell released histamine's role in over sensitizing the neuronal stimulus and further recruitment of leukocytes within ES, that was dampened with the use of H1-As (although in mice). Supported by an *in vitro* study by Van den Eynde et al. (97), which showed that G2 H1-As (Loratadine) suppressed the proliferation of endometrial stromal cells in mice, García-Izquierdo et al. (98) quoted H1-As as a potential future avenue for ES management. Furthermore, a recent study (also in mice) by Mao et al. (99) demonstrated the utility of another H1-As (Meclizine) in reducing lesional growth and endometrial fibrosis in Adenomyosis, a different entity but one that shares common pathways with ES. Simultaneously, recent literature has indeed identified H1-As as having promising potential in cancer therapy [see review by Faustino-Rocha et al. (100)]. For example, Fritz et al. (101, 102) showed that the use of Desloratadine yielded improved survival in various immunogenic cancers (including breast cancer).

As such, investigating the use of H1-As within the context of ES through a range of experiments (*in vivo* mouse models and *in vitro* cultured tissues, as well as population-wide analyses), is highly warranted. Doing so will illuminate insights into their impact at macroscopic (symptomatic), cellular, and gene-expression levels.

### 7.4 Broader considerations and implications

Several G1 and G2 H1-As have been approved by the drug regulatory bodies worldwide for their use across various pathological conditions. G2 H1-As (such as [Des]loratadine, [Levo]cetirizine, etc.) are widely available worldwide at an accessible cost, even in low-income countries. Furthermore, the safety profiles of a wide variety of H1-As have been thoroughly explored, with G2 H1-As demonstrating an excellent safety profile characterized by minimal-to-no cardiac and neurological side effects.

ES exerts a significant societal burden on the gynecological health of individuals within childbearing age, as it is considered one of the primary reasons for infertility, supported by studies finding a significantly higher incidence (up to ~50%) of patients with ES with infertility (3). Additionally, it is worth highlighting and considering the common pathways between ES and EC, as well as supportive evidence for an increased risk of EC and Ovarian (endometrioid) cancer within ES patients (103, 104).

As such, H1-As in synergistic use with existing hormonal-based therapies, if demonstrated to be efficient, could make a significant positive world-wide impact by: (1) being very cost-effective and accessible to the public; (2) potentially helping combat infertility from the perspective of efficient ES management; (3) potentially decreasing the risks of developing downstream cancers.

## Data Availability

The original contributions presented in the study are included in the article/supplementary material, further inquiries can be directed to the corresponding author.
